# Chemical Composition, Antioxidant and Antibacterial Activities of Essential Oil Obtained from Chincho (*Tagetes elliptica* Sm) Leaves Grown in the Peruvian Andes

**DOI:** 10.3390/foods12040894

**Published:** 2023-02-20

**Authors:** Francis Cerrón-Mercado, Jose Angel Perez-Alvarez, Diana Nolazco-Cama, Bettit Salva-Ruíz, Lena Tellez-Monzon, Juana Fernández-López, Manuel Viuda-Martos

**Affiliations:** 1 IPOA Research Group, Agro-Food Technology Department, Centro de Investigación e Innovación Agroalimentaria y Agroambiental (CIAGRO-UMH), Miguel Hernández University, 03312 Orihuela, Alicante, Spain; 2 Departamento de Tecnología de Alimentos y Productos Agropecuarios (TAPA), Universidad Nacional Agraria la Molina, UNALM, Lima 15024, Peru; 3 Centro de Investigación en Química, Toxicología y Biotecnología Ambiental del Departamento Académico de Química de la Facultad de Ciencias de la UNALM, Lima 15024, Peru

**Keywords:** *Tagetes elliptica* Sm., antioxidant, antibacterial, essential oil, aromatic plants

## Abstract

The chemical composition, antioxidant, and antibacterial properties of the essential oil from the leaves of *Tagetes elliptica* Sm., grown in Peru, were studied. The EO was extracted using steam distillation, and its chemical composition was analyzed using GC-MS, while the antioxidant activity was evaluated using the radical, scavenging capacity (DPPH and ABTS assays), and ferric reducing antioxidant power (FRAP) assays, ferrous ion chelating (FIC) activity, and the Rancimat test. The antibacterial activity against *Staphylococcus aureus*, *Escherichia coli*, and *Salmonella infantis* was studied using the agar well diffusion method. Twenty-seven compounds were identified in the essential oil, and the major components were *cis*-tagetenone (37.27%), *trans*-tagetenone (18.84%), dihydrotagetone (14.38%), and trans-tagetone (5.15%). With regard to antioxidant properties, the IC_50_ values obtained for the DPPH, ABTS, and FIC assays were 53.37, 46.38, and 22.65 mg/mL, respectively. These values were lower than those obtained for standard butylated hydroxytoluene and ascorbic acid. In the Rancimat test, antioxidant activity was achieved only at high concentration. *T. elliptica* essential oil showed a marked antibacterial activity against all bacterial strains at all concentrations assayed. This study demonstrated that *T. elliptica* essential oil could be considered as an alternative to synthetic antioxidants and antimicrobial agents in the food industry.

## 1. Introduction

The consumption of “clean label” foods formulated with natural ingredients has increased in recent years due to the increasing awareness among the population that a healthier lifestyle can improve their health, hence the demand for the food industries to innovate, look for alternatives, and use natural preservatives of plant origin to produce healthy, safe, tasty, and socially accepted foods [1,2]. Accordingly, in the food industry there is a tendency to decrease the use of synthetic additives that could be harmful to health [3]. In this sense, several studies have shown that aromatic herbs and spices, which are an important source of bioactive compounds, might be used as natural preservatives in the development of food additives or natural food ingredients due to their antioxidant and antimicrobial properties [1,4,5]. Additionally, these aromatic herbs and spices provide aroma and flavor to food and enhance the pleasure of eating.

The genus *Tagetes* belongs to the Asteraceae family and is represented by more than 30 species, which are adapted to live from sea level to high altitudes. It is distributed mainly in the central and southern regions of America and naturalized in many regions of Africa, Asia, and Europe [6]. These plants contain a high number of bioactive compounds, including polyphenolic, carotenoids, and terpenes that have biological properties such as antibacterial, antioxidant, antiviral, and anticancer activities, among others [7,8,9,10,11,12]. In addition, the leaves and flowers of these plants have been used to prepare infusions in folk medicine, due to their anti-inflammatory and digestive properties, as well as their properties as an analgesic to treat various ailments and relieve pain [13]. Several species of the genus *Tagetes*, including *Tagetes lucida*, *Tagetes minuta*, *Tagetes pusilla*, *Tagetes erecta*, and *Tagetes patula*, are often used as ornamental plants; however, in recent years these plant are being analyzed for their pharmaceutical activities based on their utilization in traditional medicine [14,15]. Another very important species is *Tagetes elliptica* Sm., which is an ornamental and cultivated plant that grows throughout Peru between 1000 and 4500 m above sea level in the Andean regions of Ayacucho, Junín, Ancash, and in the temperate climates of the Amazon and Lima regions. It is also well known in Central and South America [16]. In these regions, *T. elliptica* is known as “chincho”, “chinchu”, or “chikchimpa”, and it is used in traditional gastronomy in the preparation of various stews and roasts due to its characteristic aroma and flavor [17]. Additionally, *T. elliptica* leaves have been used as a natural medicine in infusions against stomach and intestinal pains [7], due to the high content of essential oil [8]. Essential oils are natural and volatile extracts that contain bioactive compounds, besides giving flavor to food [18]. In the food industry, the essential oils are generally utilized as natural preservatives for their antioxidant, antibacterial, and antifungal activity [19], increasing the quality, safety, and shelf life of food [20]. The essential oils from plants of the genus *Tagetes* have a high content in terpenes and sesquiterpenes, including β-ocimene, β-terpinene, myrcene, tagetones, dihydrotagetone, and tagetenones [14]. In addition, other studies report that the main constituents of *Tagetes* spp. essential oil are ocimenones (Z) and (E), along with piperitone, piperitenone, limonene, tagetone, and caryophyllene [21,22].

The scientific literature has revealed the beneficial effects of essential oils obtained from plants of the genus *Tagetes* as an antioxidant and antibacterial, but there are very few published works specifically investigating the bioactive properties of essential oil obtained from *T. elliptica*. Therefore, the objective of this study was to establish the chemical composition as well as the antioxidant and antibacterial activities of the essential oil obtained from the leaves of *Tagetes elliptica* Sm.

## 2. Materials and Methods

### 2.1. Plant Material and Essential Oil Extraction

**Figure 1:**
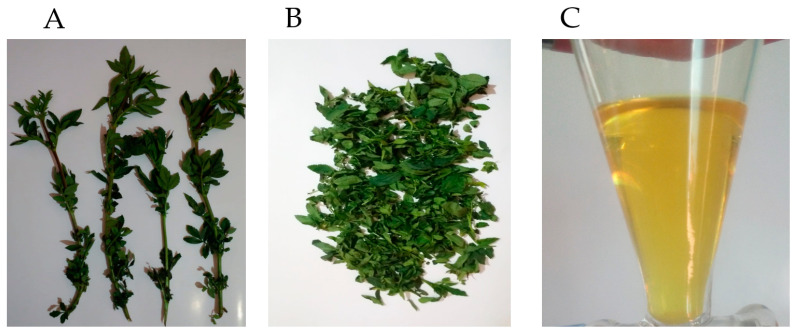
*Tagetes elliptica* (**A**) Stems and leaves; (**B**) Leaves; (**C**) Essential oil.

The leaves of *Tagetes elliptica* Sm. (Figure 1A,B) were collected in the province of Chupaca, Junín Region, Peru (3.263 m above sea level; 12°3′42″ S; 75°17′16″ W), and 500 g were hydrodistilled for 3 h using a Clevenger type apparatus to obtain the essential oil (EO). The essential oil (Figure 1C) was dried with anhydrous sodium sulfate (500 mg) and kept in sealed amber glass vials at 4 °C until further analysis.

### 2.2. Chromatographic Analysis

The chemical composition of *T. elliptica* Sm. essential oil was assessed using Hewlett-Packard 6890 gas chromatography equipment (Agilent, Santa Clara, CA, USA) equipped with a flame ionizer detector (FID) and a DB-5MS column (60 m × 250 μm, 0.25 μm film thickness). The temperature of the injector was 300 °C, whilst the temperature of detector was set at 325 °C. The oven temperature was held at 45 °C for 4 min; increased to 200 °C at 2.5 °C/min and kept for 2 min; then raised to 300 °C at 2.5 °C/min and kept for 5 min using helium as a carrier gas at 1 mL/min. Twenty μL of *T. elliptica* essential oil was diluted with 0.2 mL of GC-grade dichloromethane. One microliter of the diluted oil was then injected using an automatic injector into the system with a split ratio of 1:20. Quantitative data were obtained electronically from FID area data without using correction factors. All tests were performed in triplicate.

The *T. elliptica* essential oil was also analyzed by GC-MS with Hewlett-Packard 5975C GC-MS equipment (Agilent, Santa Clara, CA, USA). The GC-MS equipment was equipped with the same column used in the GC analysis and with the identical temperature conditions following the methodology proposed by Alves-Silva et al. [23]. The compounds were identified using the Kováts Index in reference to n-alkanes (C_8_-C_32_), with the mass spectra of authentic standards as well as with the Wiley spectral library collection.

### 2.3. Antioxidant Analysis

#### 2.3.1. DPPH Radical Scavenging Method

The 2,2-Diphenyl-2-picrylhydrazyl (DPPH) scavenging activity assay was utilized, following the methodology reported by Brand-Williams et al. [24], to determine the antioxidant activity. For that, methanolic solutions of *T. elliptica* essential oil of six different concentrations (5, 10, 20, 50, 80 and 100 mg/mL) were assessed. Butylated hydroxytoluene and ascorbic acid (0.005, 0.01, 0.02, 0.05, 0.08 and 0.1 mg/mL) were used as standards for comparison of the antioxidant potential of the essential oil. The inhibition percentage of the DPPH radical was calculated according to Equation (1).

(1) $$\%\;Inhibition=\frac{Absorbance\;of\;control\;sample-Absorbance\;of\;tested\;sample}{Absorbance\;of\;control\;sample}\times 100$$

Additionally, inhibition (%) was plotted against the essential oil concentration in the reaction system and the concentration of essential oil required to scavenge 50% of DPPH free radicals (IC_50_ value) was calculated graphically.

#### 2.3.2. ABTS Radical Scavenging Method

The methodology described by Thaipong et al. [25] was used for the determination of 2,2-Aazino-bis(3-ethylbenzthiazoline-6-sulphonic acid) using a radical scavenging assay. The methanolic solutions of *T. elliptica* essential oil, as well as butylated hydroxytoluene and ascorbic acid, were the same as those described in Section 2.3.1. The antioxidant ability of samples was expressed as the inhibition percentage of the ABTS radical cation scavenging activity following Equation (2).

(2) $$\%\;Inhibition=\frac{Absorbance\;of\;control\;sample-Absorbance\;of\;tested\;sample}{Absorbance\;of\;control\;sample}\times 100$$

The IC_50_ values were determined from a graph plotting the % inhibition against the sample concentration in the reaction system.

#### 2.3.3. Ferrous Ion Chelating (FIC) Ability

The ferrous ion chelating activity of the different concentrations of (5–100 mg/mL) of *T. elliptica* essential oil was determined following the methodology described by Sudha et al. [26]. Ethylenediaminetetraacetic acid (EDTA) at different concentrations (0.005–0.1 mg/mL) was used as a standard for comparison of the antioxidant potential of essential oil. The ferrous ion chelating activity was calculated using Equation (3).

(3) $$chelating\;effect\;\left(\%\right)=\frac{\left(1-Absrbance\;of\;sample\right)}{Absorbance\;of\;blank}\times 100$$

The IC_50_ values were determined from a graph plotting the % inhibition against the sample concentration in the reaction system.

#### 2.3.4. Ferric Reducing Antioxidant Power (FRAP)

The ability to reduce ferric ions of methanolic solutions of *T. elliptica* essential oil at six different concentrations (0.3125, 0.625, 1.25, 3.125, and 5 mg/mL) as well as butylated hydroxytoluene and ascorbic acid (0.0625, 0.125, 0.25, 0.625, and 1 mg/mL) was assessed using the methodology described by Dudonné et al. [27]. The FRAP values were expressed in terms of Trolox equivalent antioxidant capacity (TEAC) in mM Trolox/L.

#### 2.3.5. Rancimat Assay

The Rancimat test was used to determine the antioxidant ability of *T. elliptica* Sm. essential oil, at different concentrations (5, 10, 20, 50, 80 and 100 mg/mL) against melted pork butter following the method described by Viuda-Martos et al. [28]. Butylated hydroxytoluene and ascorbic acid (0, 10, 20, 50, 80 and 100 mg/mL) were used as standards for comparison to the antioxidant potential of essential oil. The antioxidant activity index (AAI) was determined from the measured induction times, following the methodology described by Forster et al. [29] according to Equation (4).

(4) $$AAI=\frac{induction\;period\;of\;lard\;with\;antioxidant}{induction\;period\;of\;pure\;lard}$$

An antioxidant activity index higher than 1 indicates inhibition of the lipid oxidation; the higher the value, the better the antioxidant activity [30].

### 2.4. Antibacterial Activity

#### 2.4.1. Microbial Strains

The *T. elliptica* EO was individually tested against *Staphylococcus aureus ATCC 25923TM, Escherichia coli ATCC 25922TM*, and *Salmonella infantis*. The bacterial strains were cultured overnight in nutrient broth at 37 °C until a suspension of 1.0 × 10^7^ CFU/mL was attained.

#### 2.4.2. Agar-Well Diffusion Method

The antibacterial activity of *T. elliptica* EO was assessed using the agar-well diffusion method following the recommendations of Tepe et al. [31]. The bacterial inoculum (100 μL of 10^7^ CFU/mL) was spread homogeneously with a sterile Digralsky loop on a Mueller Hinton agar Petri dish. A hole (6 mm in diameter) was aseptically perforated on the agar surface with a sterile tip, and 40 μL of *T. elliptica* EO was added into the well. After incubation for 24 h at 37 °C, all Petri dishes were observed for any zones of growth inhibition, and the diameters of these zones were measured in millimeters.

#### 2.4.3. Determination of Concentration Effect

The effect of concentration (CE) was analyzed to determine which volume of *T. elliptica* EO showed an inhibiting effect on bacterial growth using the agar-well diffusion method. The same methodology described in Section 2.4.2 was used but adding 20, 10, and 5 mL of *T. elliptica* EO in each well, representing volumes 50%, 25%, and 12.5% less of the initial volume [32].

### 2.5. Statistical Analysis

All experiments were conducted in triplicate and the data were presented as mean ± standards deviation of triplicate determinations. The results obtained for antioxidant and antibacterial properties were examined by means of a univariate technique GLM (General Linear Model). For antioxidant and antibacterial activities, an ANOVA was applied and Tukey’s post hoc test was used (*p* < 0.05) for the comparison between means. All determinations were analyzed using SPSS^®^ Statistics 22.0.0.0. software (IBM Corp., Armonk, NY, USA).

## 3. Results and Discussion

### 3.1. Chemical Composition of Essential Oil

The essential oil obtained from the leaves of *T. elliptica Sm.* had a bright light-yellow color with a pleasant odor. The yield of the essential oil recorded on a fresh weight basis was 0.23% (*w*/*w*). These values were lower than those reported for plants of the genus *Tagetes* cultivated in Iran (0.57 and 0.48% *w*/*w*) or in the Kingdom of Saudi Arabia (0.84% *w*/*w*) [33,34]. This variation could be due to several factors including species, part of the part used to obtain the essential oil, or environmental conditions, among others.

In the GC/MS analysis of the *T. elliptica* essential oil, twenty-seven compounds were identified representing 96.8% of the total essential oil (Table 1). The main constituents were *cis*-tagetenone (37.27%), *trans*-tagetenone (18.84%), dihydrotagetone (14.38%), and *trans*-tagetone (5.15%).

To our knowledge, there are no studies in which the chemical composition of *T. elliptica* essential oil has been determined. However, several studies about the chemical composition of essential oils obtained from others *Tagetes* species have been widely reported. In this sense, Kyarimpa et al. [35] analyzed the essential oil obtained from the aerial part of *Tagetes minuta* grown in Uganda. These authors reported that the main constituents of this essential oil were *trans*-ocimene (15.90%), I-verbenone (15%), limonene (8.02%), and tagetone (3.56%). In a similar work, Hartwig de Oliveira et al. [9] carried out a study to analyze the chemical composition of *T. minuta* essential oil obtained from flowers cultivated in Brazil. They found that the principal components of this essential oil were (Z)-Tagetone (70.64%), β-ocimene (11.18% *w*/*w*), and (E)-tagetone (6.24%). Omer et al. [36] investigated the chemical composition of essential oil obtained from *Tagetes lucida* cultivated in Egypt. These authors reported that the main constituents of this essential oil were methyl chavicol (93.18%) and linalool (2%). Gakuubi et al. [37] reported that the most abundant compounds identified in the essential oils obtained from aerial parts at the flowering stage of *T. minuta* cultivated in Kenya were (E)-tagetone, dihydrotagetone, and allo-ocimene. More recently, Moghaddam et al. [33] investigated the chemical composition of the essential oils obtained from leaves and flowers of *Tagetes patula* L. and *Tagetes erecta* L. grown in Iran. The main compounds found in *T. patula* essential oil were β-caryophyllene (24.53%), piperitenone (10.96%), and piperitone (9.66%), whilst the *T. erecta* essential oil was characterized by neophytadiene (17.22%), piperitone (12.52%), and β-caryophyllene (8.7%). Aati et al. [34] reported that the principal components of essential oil from *T. patula* cultivated in the Kingdom of Saudi Arabia were β-caryophyllene (24.1%), 2-undecanone + bornyl acetate (12.2%), and 2-nonanone (9.7%).

The scientific literature shows that the quantitative ratios of the oil components of essential oils obtained from plants of the genus *Tagetes* ssp. depend on several factors, including the species, crop growing location, plant development stage, harvest period, cultivation practices, parts of plant used for oil isolation, and environmental conditions, among others [38].

### 3.2. Antioxidant Activity of T. elliptica Essential Oil

In general terms, the antioxidant properties of essential oils could differ depending on several factors including the chemical profile and the methods assessed. In this research, five methodologies (DPPH assay, ABTS assay, FRAP assay, FIC assay, and Rancimat test), with different action mechanisms, were used. These methodologies have been widely utilized to estimate the antioxidant capacity of several products. The DPPH assay assesses the ability of phytochemicals to donate hydrogen atoms to the DPPH radical, which causes a color change in the DPPH solution. The greater the color change, the greater the antioxidant capacity, which is represented by a lower IC_50_ value. Table 2 shows the antioxidant properties of *T*. *elliptica* essential oil, as well as the butylated hydroxytoluene (BHT) and ascorbic acid (AA) tested with the DPPH radical scavenging assay at different concentrations. As can be see, a concentration-dependent scavenging activity (*p* < 0.05) was found for *T*. *elliptica* essential oil, as well as for BHT and AA. However, it is important to highlight that the concentrations tested for the positive controls (BHT and AA) were 1000 times lower than those used for *T*. *elliptica* essential oil. This fact it is reflected in the IC_50_ values (*p* < 0.05), which were 53.37, 0.17, and 0.02 mg/mL for *T*. *elliptica* essential oil, BHT, and AA, respectively.

The IC_50_ values obtained in this work were higher than those reported by Ruiz et al. [39] for the essential oils obtained from aerial parts of *T. elliptica* cultivated in Peru (IC_50_ = 3.4 mg/mL), as well as for the essential oils of *T. minuta* and *Tagetes filifolia* with IC_50_ values of 0.8 and 20.2 mg/mL, respectively. In a similar work, Huaraca-Aparco et al. [8] reported that IC_50_ values of essential oils obtained from *T. elliptica* and *T. minuta* cultivated in Peru analyzed with a DPPH assay were 2.56 and 1.77 mg/mL, respectively. Ali et al. [40] reported that essential oil obtained from the aerial parts of *T. minuta* cultivated in Yemen had an IC_50_ value of 0.04 mg/mL. Similarly, Kyarimpa et al., [41] reported that the essential oils obtained from aerial parts of *T. minuta* cultivated in Uganda exhibited strong antioxidant activities when measured with a DPPH assay. With regard to the ABTS assay, significant free radical scavenging activity was observed at all concentrations of *T. elliptica* essential oil, and for BHT and AA, as shown in Table 2. This reduction in free radical scavenging occurs in a concentration-dependent way (*p* < 0.05). The IC_50_ values (*p* < 0.05) calculated with this methodology were 46.38, 0.016, and 0.017 mg/mL for *T*. *elliptica* essential oil, BHT, and AA, respectively. It is important to highlight that the IC_50_ values achieved with the ABTS assay were less than those obtained with the DPPH assay. These variations between the two methodologies used may be explained by the fact that they have different action mechanisms. As mentioned by Platzer et al. [42], in the ABTS assay the reaction involves e^−^ transfer and occurs at a much faster speed compared with DPPH radicals, whose reaction could be attributed to the hydrogen donating capacity of several compounds present in *T. elliptica* essential oil. As occurs in the DPPH assay, the IC_50_ values obtained in the ABTS assay for *T. ellipitica* essential oil were higher than those reported by Huaraca-Aparco et al. [8], who reported that the IC_50_ values for *T. elliptica* and *T. minuta* essential oils were 41.06 and 21.02 mg/mL. On the other hand, Hartwig de Oliveira et al. [9] reported that the IC_50_ value attained in the ABTS assay for *T. minuta* essential oil obtained from flowers cultivated in Brazil was 0.1 mg/mL.

Other mechanism of antioxidant action is the chelation of transition metals. These metals may promote the lipid peroxidation via the generation of initiator species and increasing peroxidation. Thus, the study of the metal ion-chelating properties (Table 2) showed that *T. elliptica* essential oil was capable of chelating Fe^+2^ when high concentrations (12.5–25 mg/mL) were assayed, with statistical differences (*p* < 0.05) between all samples. At low concentrations (1.25–5 mg/mL), no chelating effect was observed. Similarly, at low concentrations (0.005–0.02 mg/mL) of EDTA used as standard, no chelating activity was observed. The IC_50_ values for *T. elliptica* essential oil and positive control EDTA were 22.65 and 0.06 mg/mL, respectively.

The ferric reducing properties of *T. elliptica* essential oil, BHT, and AA were attained utilizing the FRAP methodology as shown in Table 3. As can be seen, the ferric reducing capacity for all samples assayed occurred in a concentration-dependent manner. These results agree with those reported by Hartwig de Oliveira [9], who reported that the essential oil obtained from flowers of *T. minuta* showed a reducing potential in a concentration-dependent manner possibly due to an electron transfer mechanism. Mlala et al., [43] reported that the essential oil obtained from leaves of *T. minuta* cultivated in South Africa had a ferric reducing capacity of 63.56 µg ascorbic acid equivalent/mL of extract.

Rancimat assay is a cheap and simple methodology that needs low sample volumes and attains reproducible results. Table 4 shows the values obtained for the antioxidant activity index (AAI) of animal fat with *T. elliptica* essential oil added, and with the standard BHT and AA essential oils added. Thus, the longer the induction period of the animal butter added with the essential oil, BHT, or AA standards compared to the control, which is pure butter, the more potent the antioxidant capacity of that compound will be. The AAI decreased in the order AA > BHT > *T. elliptica* Sm. According to this methodology, only concentrations of 5 and 10 mg/L of *T. elliptica* Sm. essential oil displayed antioxidant activity, with AAI values of 1.01 for both concentrations. These values were lower than those obtained for standard compounds (Table 4). It is important to highlight that *T. elliptica* Sm. essential oil showed pro-oxidant effects at concentrations ranging between 20 and 100 mg/mL). The pro-oxidant effect of essential oils is widely described in the scientific literature, as described in the works of Poma et al. [44] and Kong et al. [45].

The results obtained show that the essential oils obtained from aerial parts of *T. ellipitica* had a moderated antioxidant activity. These antioxidant properties could be attributed to a high content of acyclic monoterpenes ketones, including *cis*- and *trans*-tagetenone and tagetone, found in their composition as well as the synergistic action among several major and minor components [21,46]. However, the mechanism of action by which the compounds present in essential oils exert their antioxidant effect is not yet clearly understood. In this way, several mechanisms have been proposed, principally their redox properties, that play a significant role in absorbing and neutralizing free radicals, quenching singlet oxygen, and decomposing peroxides, as reported Jugreet et al. [47].

### 3.3. Antibacterial Activity of T. elliptica Essential Oil

The agar-well diffusion method was used to analyze the antibacterial activity of *T. elliptica* essential oil against Gram-positive and Gram-negative bacteria as shown in Table 5. These bacterial strains, *S. aureus* and *E. coli*, are considered model pathogenic bacteria for the evaluation of antimicrobial activity of plant extracts [48] while *S. infantis* is a pathogenic bacterium responsible for zoonoses transmissible to humans, with high resistance to antibiotics.

*T. elliptica* EO showed antibacterial activity against all strains studied. Thus, *Staphylococcus aureus* (Gram-positive) had the highest inhibition halo (*p* < 0.05), while *E. coli* and *S. infantis* showed lower inhibition diameters with no difference (*p* > 0.05) between them. To the best of our knowledge, this is the first work to determine the antibacterial properties of the essential oil obtained from leave of *T. elliptica.* Nevertheless, as occurs with the chemical composition, the antibacterial properties of several other species of the genus *Tagetes* have been widely studied. Thus, Shirazi et al. [49] assessed the antibacterial activity of essential oils obtained from the leaves and flowers of *Tagetes minuta* cultivated in Iran. These authors reported that the minimal inhibitory concentration against *Salmonella typhi*, *Escherichia coli*, *Staphylococcus aureus*, and *Bacillus subtilis* were 150, 165, 67, and 75 μg/mL of *T. minuta EO*, respectively. Similarly, Gakuubi et al. [37] reported that the essential oil of *T. minuta* obtained from the leaves, flowers, and stem (at the flowering stage), with a volume added to the disk of 10 µL, had a very important antibacterial activity against *Pseudomonas savastanoi*, *Xanthomonas axonopodis*, and *Xanthomonas axonopodis* with inhibition halos of 41.83, 26.83, and 26.83 mm, respectively. More recently, Safar et al. [50] reported antibacterial activity of *Tagetes patula* EOs against several bacterial strains, including *Serratia fonticola*, *Klebsiella pneumoniae*, *Proteus mirabilis*, *Escherichia coli*, and *Staphylococcus aureus*, with minimum inhibitory concentration values ranging between 0.16 and 0.64 µL/mL.

The results obtained in this work showed that *T. elliptica* essential oil was more effective against Gram-positive bacteria when compared to Gram-negative. In this sense, several studies on the antibacterial properties of essential oils obtained from plants of the genus *Tagetes*, as well as other plant species, have commonly reported that the Gram-positive bacteria were more susceptible to the effects of EOs in comparison to Gram-negative bacteria [49,51,52] due to the fact that the Gram-negative bacteria have an outer membrane that restricts the diffusion of hydrophobic substances [53]. In view of the huge number of diverse groups of chemical compounds found in *T. elliptica* essential oil, it is most likely that their antibacterial activity is not attributable to one specific compound. In addition, the mechanism of action of essential oils obtained from plants of the genus *Tagetes* is still unknown. Several studies have reported that the cell membrane is the principal structure of the microorganism which is damage by the most of chemical compounds found in essential oils [54]. In addition, several chemical compounds found in essential oils may change the successive process of the synthesis of macromolecules, including proteins, polysaccharides, DNA, or RNA, which finally provoke the cell’s death [55]. Therefore, Senatore et al. [56] reported that that terpenoids such as dihydrotagetones, tagetones, and ocimenones, which were found in *T. elliptica* essential oils, are sufficient to account for the observed antibacterial activities of this essential oil due to the fact that they may provoke several impairments to the cell membrane, including some distorted projections on the cell membrane, and the cell morphology.

The concentration effect of the essential oil from *T. elliptica* on the three bacterial strains studied is shown in Table 5. All the concentrations assayed produced inhibition halos against all bacterial strains. In the case of *S. aureus*, this fact occurs in a concentration-dependent manner (*p* < 0.05). However, in the case of *E. coli* and *S. infantis*, no statistical differences were found between any of the concentrations analyzed.

As mentioned in Ruiz-Navajas et al. [32] it is important to notice that, when the essential oils are utilized as antimicrobial agents, their effectiveness is reduced when these phytochemicals are incorporated into complex matrices such as foods, without forgetting the impact on the organoleptic properties may have. In addition, several questions about toxicity and safety should be taken into consideration.

## 4. Conclusions

The results obtained in this work demonstrated that *T. elliptica* Sm. essential oil is a good source of important bioactive compounds (*cis*-tagetenone, *trans*-tagetenone, and dihydrotagetone) and possesses a moderate antioxidant capacity with different possible action mechanisms. In the same way, this essential oil has strong antibacterial properties against both Gram-negative and Gram-positive bacteria. However, further studies would be necessary to clarify the compound or compounds responsible for both antioxidant and antibacterial activity.

In any case, in view of these properties, this essential oil could be considered a safe and eco-friendly alternative to synthetic antioxidants and antimicrobial agents in the food industry. Despite the promising results obtained in in vitro assays, more detailed studies of the mechanisms of action of *T. elliptica* essential oils would be beneficial to achieve their potential as a natural preservative in the development of food products. This is because, when the essential oils are added to more complex matrices such as food, they tend to reduce both antioxidant and antibacterial properties due to possible interactions between food and essential oil components. This would make it necessary to add higher concentrations to achieve the same effect, which could have undesirable effects on the organoleptic characteristics of the product.

## Figures and Tables

**Table 1:** Chemical composition of *T. elliptica* essential oil obtained from leaves.

No	Compound	Retention Time	Kovats Index	Area (%)
1	*α*-pinene	13.28	936	0.66 ± 0.01
2	*β*-mircene	14.53	986	0.87 ± 0.02
3	*β*-Pinene	14.91	990	0.62 ± 0.02
4	*α*-phellandrene	15.62	1005	1.71± 0.04
5	*β*-*trans*-ocimene	16.40	1022	4.66 ± 0.11
6	*β*-phellandrene	16.46	1029	0.22 ± 0.01
7	eucalyptol	16.51	1031	0.47± 0.01
8	dihydrotagetone	16.98	1045	14.38± 0.21
9	*β*-linalool	18.48	1098	0.45± 0.01
10	allo-ocimene	18.88	1131	0.50 ± 0.01
11	cis-epoxyocimene	18.97	1134	3.62 ± 0.04
12	trans-epoxyocimene	19.35	1140	0.85 ± 0.00
13	(*Z*) *trans*-tagetone	19.91	1143	5.15 ± 0.00
14	(*E*) *cis*-tagetone	20.11	1146	3.42 ± 0.06
15	p-cymen-8-ol	20.35	1171	0.59 ± 0.07
16	*α*-terpineol	21.61	1187	0.19 ± 0.01
17	decanal	21.72	1202	0.98 ± 0.01
18	verbenone	21.86	1214	0.97 ± 0.07
19	(*E*) *cis*-Tagetenone	22.64	1231	37.27 ± 0.24
20	(*Z*) *trans*-Tagetenone	22.84	1250	18.84 ± 0.29
21	anisole	23.28	1264	0.40 ± 0.01
22	piperitone	23.82	1281	0.40 ± 0.00
23	*β*-caryophyllene	28.10	1436	0.97 ± 0.00
24	germacrene D	29.71	1491	0.45 ± 0.01
25	biciclogermacrene	30.10	1510	0.46 ± 0.00
26	guaiol	31.42	1598	0.36 ± 0.00
27	*α*-cadinol	32.37	1656	0.54 ± 0.02

**Table 2:** Antioxidant activity of *T. elliptica* essential oil measured by DPPH, ABTS and FIC assays.

**DPPH Assay**				
*T. elliptica* essential oil			BHT	AA
Concentration (mg/mL)	% Inhibition of DPPH radical	Concentration (mg/mL)	% Inhibition of DPPH radical	% Inhibition of DPPH radical
5	4.90 ± 2.36 ^a^	0.005	4.25 ± 0.18 ^a^	13.63 ± 2.78 ^a^
10	11.17 ± 2.15 ^b^	0.01	6.29 ± 0.79 ^b^	23.54 ± 2.83 ^b^
20	21.14 ± 1.78 ^c^	0.02	10.17 ± 1.52 ^c^	56.36 ± 1.85 ^c^
50	48.09 ± 1.60 ^d^	0.05	17.50 ± 0.40 ^d^	96.01 ± 0.17 ^d^
80	77.77 ± 2.85 ^e^	0.08	21.26 ± 0.21 ^e^	96.26 ± 0.23 ^d^
100	87.63 ± 0.57 ^f^	0.10	33.21 ± 2.59 ^f^	96.33 ± 0.13 ^d^
*IC_50_ (mg/mL)	53.37 ± 1.43	*IC_50_ (mg/mL)	0.17 ± 0.01	0.02 ± 0.00
**ABTS Assay**				
*T. elliptica* essential oil			BHT	AA
Concentration (mg/mL)	% Inhibition of ABTS radical	Concentration (mg/mL)	% Inhibition of ABTS radical	Concentration (mg/mL)
5	8.10 ± 0.85 ^f^	0.005	28.26 ± 2.01 ^f^	22.55 ± 2.01 ^f^
10	12.89 ± 1.51 ^e^	0.01	42.05 ± 0.68 ^e^	45.51 ± 0.58 ^e^
20	24.01 ± 0.81 ^d^	0.02	62.03 ± 0.63 ^d^	62.25 ± 0.22 ^d^
50	48.96 ± 0.97 ^c^	0.05	73.18 ± 0.20 ^c^	78.89 ± 0.18 ^c^
80	68.95 ± 1.59 ^b^	0.08	84.48 ± 1.90 ^b^	86.17 ± 0.23 ^b^
100	86.98 ± 0.58 ^a^	0.10	92.91 ± 1.40 ^a^	99.18 ± 0.09 ^a^
*IC_50_ (mg/mL)	46.38 ± 2.16	*IC_50_ (mg/mL)	0.016 ± 0.00	0.017 ± 0.001
**FIC Assay**				
*T. elliptica* essential oil				EDTA
Concentration (mg/mL)	Chelating effect (%)		Concentration (mg/mL)	Chelating effect (%)
1.25	---		0.005	---
2.50	---		0.01	---
5	---		0.02	---
12.50	27.48 ± 2.87 ^c^		0.05	41.47 ± 2.95 ^c^
20	39.90 ± 1.44 ^b^		0.08	71.92 ± 3.46 ^b^
25	58.92 ± 0.98 ^a^		0.10	80.22 ± 1.82 ^a^
*IC_50_ (mg/mL)	22.65 ± 0.80		*IC_50_ (mg/mL)	0.06 ± 0.00

Values are expressed as mean ± SD of three independent experiments. *IC_50_: concentration of essential oil (mg/mL) for a 50% inhibition. EDTA: Ethylenediaminetetraacetic acid. For the same method, (DPPH, ABTS, and FIC) values followed by the different letter within the same column are significantly different (*p* < 0.05) according to Tukey’s Multiple Range Test.

**Table 3:** Antioxidant activity of *T. elliptica* essential oil measured by FRAP method.

*T. elliptica* Essential Oil			BHT	AA
Concentration (mg/mL)	TEAC* mMTrolox/L	Concentration (mg/mL)	TEAC* mMTrolox/L	TEAC* mMTrolox/L
0.3125	0.06 ± 0.01 ^a^	0.0625	0.17 ± 0.01 ^a^	0.03 ± 0.0 ^a^
0.625	0.23 ± 0.02 ^a^	0.125	0.49 ± 0.02 ^a^	0.07 ± 0.0 ^a^
1.25	0.79 ± 0.04 ^a^	0.25	1.25 ± 0.05 ^b^	0.15 ± 0.01 ^b^
3.125	2.32 ± 0.42 ^b^	0.625	4.44 ± 0.17 ^c^	0.43 ± 0.00 ^c^
5	8.73 ± 0.53 ^c^	1	7.97 ± 0.21 ^d^	0.67 ± 0.05 ^d^

TEAC*: Trolox equivalent antioxidant capacity. BHT: Butylated hydroxytoluene, AA: ascorbic acid. Values are expressed as mean ± SD of three independent experiments. Values followed by the different letter within the same column are significantly different (*p* < 0.05) according to Tukey’s Multiple Range Test.

**Table 4:** Antioxidant activity of *T. elliptica* essential oil measured by Rancimat test.

	Antioxidant Activity Index (AAI)		
Concentration (mg/mL)	*T. elliptica* Essential Oil	BHT	AA
5	1.01 ± 0.03 ^a^	1.09 ± 0.07 ^b^	1.51 ± 0.04 ^c^
10	1.01 ± 0.04 ^a^	1.18 ± 0.07 ^b^	1.59 ± 0.13 ^c^
20	0.99 ± 0.04 ^a^	1.28 ± 0.11 ^a,b^	1.84 ± 0.25 ^b,c^
50	0.91 ± 0.03 ^a^	1.40 ± 0.13 ^a,b^	2.12 ± 0.27 ^a,b^
80	0.95 ± 0.04 ^a^	1.55 ± 0.20 ^a^	2.36 ± 0.10 ^a^
100	0.93 ± 0.03 ^a^	1.58 ± 0.07 ^a^	2.49 ± 0.22 ^a^

BHT: Butylated hydroxytoluene, AA: ascorbic acid. Values are expressed as mean ± SD of three independent experiments. Values followed by the different letter within the same column are significantly different (*p* < 0.05) according to Tukey’s Multiple Range Test.

**Table 5:** Antibacterial activity of *T. elliptica* essential oil using the agar-well diffusion method.

		Diameter (Mean and SD) of Inhibition Zone (mm) Including Well Diameter of 6 mm		
Essential Oil	Volume (µL)	*S. aureus*	*E. coli*	*S. infantis*
*T. elliptica*	40	19.67 ± 0.57 ^a^	12.63 ± 0.56 ^a^	13.00 ± 1.00 ^a^
	20	17.33 ± 0.16 ^b^	11.19 ± 1.20 ^ab^	12.33 ± 0.58 ^ab^
	10	15.57 ± 0.35 ^c^	10.87 ± 0.85 ^b^	11.23 ± 0.61 ^b^
	5	14.30 ± 0.23 ^d^	10.00 ± 1.00 ^b^	10.63 ± 0.55 ^c^

Values followed by the same letters within the same column are not significantly different (*p* > 0.05) according to Tukey’s multiple range test.

## Data Availability

The data presented in this study are available on request from the corresponding author.
